# A Bibliometric Network Analysis of Recent Publications on Digital Agriculture to Depict Strategic Themes and Evolution Structure

**DOI:** 10.3390/s21237889

**Published:** 2021-11-26

**Authors:** Michele Kremer Sott, Leandro da Silva Nascimento, Cristian Rogério Foguesatto, Leonardo B. Furstenau, Kadígia Faccin, Paulo Antônio Zawislak, Bruce Mellado, Jude Dzevela Kong, Nicola Luigi Bragazzi

**Affiliations:** 1 Business School, Unisinos University, Porto Alegre 91330-002, RS, Brazil; consultor1@gruporara.com.br (C.R.F.); kadigiaf@unisinos.br (K.F.); 2 School of Management, Federal University of Rio Grande do Sul, Porto Alegre 90040-060, RS, Brazil; leandronascimento@ufrgs.br (L.d.S.N.); pazawislak@ea.ufrgs.br (P.A.Z.); 3 Department of Industrial Engineering, Federal University of Rio Grande do Sul, Porto Alegre 90040-060, RS, Brazil; leonardo.furstenau@ufrgs.br; 4 School of Physics and Institute for Collider Particle Physics, University of the Witwatersrand, Johannesburg 2050, South Africa; bruce.mellado@wits.ac.za; 5 Department of Mathematics and Statistics, York University, Toronto, ON M3J 1P3, Canada; jdkong@yorku.ca

**Keywords:** precision agriculture, agriculture 4.0, digital agriculture, smart farming, industry 4.0, sustainability, innovation, bibliometrics, science mapping

## Abstract

The agriculture sector is one of the backbones of many countries’ economies. Its processes have been changing to enable technology adoption to increase productivity, quality, and sustainable development. In this research, we present a scientific mapping of the adoption of precision techniques and breakthrough technologies in agriculture, so-called Digital Agriculture. To do this, we used 4694 documents from the Web of Science database to perform a Bibliometric Performance and Network Analysis of the literature using SciMAT software with the support of the PICOC protocol. Our findings presented 22 strategic themes related to Digital Agriculture, such as Internet of Things (IoT), Unmanned Aerial Vehicles (UAV) and Climate-smart Agriculture (CSA), among others. The thematic network structure of the nine most important clusters (motor themes) was presented and an in-depth discussion was performed. The thematic evolution map provides a broad perspective of how the field has evolved over time from 1994 to 2020. In addition, our results discuss the main challenges and opportunities for research and practice in the field of study. Our findings provide a comprehensive overview of the main themes related to Digital Agriculture. These results show the main subjects analyzed on this topic and provide a basis for insights for future research.

## 1. Introduction

The field and the concept itself of agriculture have dramatically changed and have been profoundly revolutionized over time, with the introduction of new approaches and concepts, such as those of Precision Agriculture (PA) and Agriculture 4.0 (A4.0). These have given rise to so-called Digital Agriculture (DA). Around 10,000 years ago, humans began to dedicate efforts to domesticating plants and animals, spreading seeds, planting seedlings, and using animal force to plow the land [1]. This revolution that changed human life and transformed hunter-gatherers into farmers is called the Neolithic Revolution or the First Agricultural Revolution [2]. Although this revolution was a great leap for humankind—as it allowed the cultivation of plants and guaranteed food for the population—the new activities demanded more hours of work [2]. This dedication to agriculture generated a surplus of food that multiplied the number of people and created elites who owned and controlled land and food [2]. Between the First Agricultural Revolution and the prosperous and abundant society that we have today, thousands of years have passed, and several transformations have occurred in the ways of production, and several difficulties related to economic niches, malnutrition and wars over fields and cultures have arisen [2]. These significant disadvantages and shortcomings have led to new transformations and revolutions in the field of agriculture [3].

In the 19th century, Agriculture 2.0, also known as the Second Agricultural Revolution, changed farming by introducing ad hoc machinery, selective breeding, animal fertilizer, crop rotation, reapers and tractors that contributed to shaping the first steps of modern agriculture [1]. Food started to be produced faster in this frame, but farmers were hindered by rust, pests, and soil depletion. New techniques and technologies have increased food surplus and reduced the need for labor in agriculture, forcing the migration of workers and the urbanization and the growth of cities [4,5]. This workforce allocation in towns was an integral part of the industrial revolution, which stimulated the rise of manufacturing industries [4].

To deal with the challenges of the Second Agricultural Revolution, Agriculture 3.0 or the Third Agricultural Revolution emerged in the 20th and early 21st centuries. Between 1966 and 1985, the concept of the Green Revolution was introduced, especially in terms of the use of advanced, cutting-edge technologies, such as biotechnology and genetic engineering to generate genetically varieties, hybrid plants and animals [6,7], and this provided a real breakthrough in agriculture. The third revolution was characterized by the further introduction of new technologies, such as GNSS (Global Navigation Satellite System) to monitor animals and crops, computers and devices for data management and analysis of agricultural production-related information, robots, chemicals, fertilizers, and irrigation systems to increase productivity and production quality [1]. These transformations greatly impacted the quality of agricultural production and the societal, economic, and environmental pillars of sustainability [8].

The concept of PA emerged in 1980 and represented the adoption of precision techniques and technologies used singly or in combination [9] to control field variability [10,11]. Despite the concept of PA appearing during the Third Agricultural Revolution, its techniques and technologies were mainly exploited in the context of the Fourth Industrial Revolution, driven by the Industry 4.0 concept [11]. A4.0 represents the use of technologies such as the Internet of Things (IoT), Artificial Intelligence, big data, cloud computing, and other smart systems and devices for managing crops and farms [11,12]. In this sense, both terms are related to DA, which can be perceived as a major driver for changes and evolution in sustainability, food security, efficiency, and productivity. 

Despite the fact that these revolutions have, at least partially, solved food production and security problems, the increase in population and the scarcity of resources are worrisome challenges for modern agriculture. Nowadays, almost one billion people go to sleep hungry every day [13]. The human population is expected to reach 9 billion by 2050 [11], increasing food demand by up to 50% [14]. Most of these people are expected to live in South Asia and sub-Saharan Africa, where more than 20% of the current population is food insecure [15]. In addition, agriculture is responsible for consuming 70% of the world’s drinking water [11]. Human consumption of resources has tripled in the last 50 years, and the exploitation of resources is 30% greater than nature’s ability to regenerate [16]. From this perspective, the challenges involved in producing more food with less environmental impact are evident [17]. Given this backdrop, emerging technologies can enable sustainable agricultural production, and the main challenges, in terms of either structural or perceived obstacles and barriers, as well as the major opportunities of their technological adoption, need to be explored.

Several studies have revised the changes and revolutions that have occurred in the field of agriculture to understand the effects of technology adoption and its potential to deal with challenges related to food production for the current and future generations. Kim et al. (2019) and Raparelli and Bajocco (2019) [18,19] performed bibliometric analyses on the use of Unmanned Aerial Vehicles (UAV) in agriculture, and Terence and Purushothaman (2020) and Navarro et al. (2020) [20,21] systematically reviewed the use of IoT in smart farms. In addition, previously published studies explored the use of Machine Learning (ML) techniques [22,23,24], geospatial technology [25], and big data for smart farming [26,27]. Extensive reviews show PA technologies’ state of the art [28] and the main factors that influence technological adoption [29]. Although some reviews and overviews have already been carried out, to the best of our knowledge, no study has investigated the complete evolution of the field of study over time or discussed the digitalization process of agriculture. The present paper addresses precisely this literature gap by highlighting the main challenges and opportunities related to Digital Agriculture.

Due to the transformational potential of emerging technologies for agriculture overviewed in the above-mentioned reviews, the present paper is devoted to offering a scientific mapping approach to identify strategic themes, the thematic evolution structure and the main challenges and opportunities of Digital Agriculture. For this, the PICOC protocol (Population, Intervention, Comparison, Outcome, and Context) was used to guide and ensure the quality of this study. The SciMAT (Science Mapping Analysis Software Tool), developed by Cobo et al. (2012) [30], was used for data treatment and generation of the strategic diagram and the evolutionary map of the field of study.

The paper is organized as follows. Section 2 presents the research background. Section 3 contains materials and methods. Results and discussions are presented in Section 4. The main challenges and opportunities are presented in Section 5, and Section 6 presents the conclusions, limitations, and suggestions for further research.

## 2. Research Background

Different terms can be used (and have been used) to refer to the fields of PA and A4.0 [31]. Some authors closely associate them, whereas, in the current work, we operate a distinction between PA and A4.0. While PA is often related to issues of field variability and specific analyses and technologies, such as application of agricultural inputs in areas with the most significant productive potential (e.g., the use of georeferenced data for the application of chemicals according to the needs of each area), A4.0 goes beyond the analysis of field variability and aims to manage farms based on detailed knowledge of the specific context and situation, to create a value chain that fully integrates, incorporates and involves stakeholders, technologies and agricultural processes [11,31,32]. Due to the variety of concepts, theoretical frameworks/approaches and interpretations related to the technological adoption in agriculture, in this research, the term Digital Agriculture is used to describe both concepts associated with digital transformation in agriculture without considering the technological level.

### 2.1. Precision Agriculture

The PA concept represents the use of techniques and emerging technologies for crop management, pest control and field improvement [11,33]. With PA, it is possible to increase crop quality and crop productivity. Since 1980, with the introduction of sophisticated, cutting-edge analyses, there has been a considerable increase in the use of technology in agriculture. For instance, the global soybean production could be 32 million tons higher if the PA were massively adopted [34]. Progressively, new techniques and technologies were developed, with researchers and practitioners gradually exploring how to provide an enhanced range of advantages for the primary sector [11].

Therefore, several studies have shown the importance of the adoption of PA for reducing/minimizing environmental impacts while preserving food production [35,36,37]. For instance, the efficient analysis of phosphorus and potassium through PA technologies contributes to agriculture being more sustainable [34]. PA research is also related to the identification of management zones [38,39], analysis and management of soil and plant nutrients [40,41,42], UAV applications for a proper capture and analysis of plantation data [43,44], farmers’ perception [45] and others.

Related to PA, there are several terms and concepts, ranging from Precision/Open Field/Farming (PF) to Precision Horticulture (PH) (or controlled-environmental agriculture (CEA) and Vertical Farming (VF)), Precision Animal Farming (PAF), including Precision Aquaculture (Fisheries). These sectors are also very important in food production, being “concentrated capital” and “economically and technologically manageable”. This reflects the non-uniform, but multi-faceted and complex nature of the field of PA. 

### 2.2. Agriculture 4.0

The concept of A4.0 emerged after the introduction and development of the concept of Industry 4.0 in 2011 and seeks to use the same technologies used by factories of the future to create a new level of agricultural production for ensuring food safety and protection of the environment [11]. A4.0 represents the use of emerging technologies to develop a value chain that successfully integrates organizations, farmers, customers, and all relevant stakeholders in favor of economic, social, and environmental sustainability. Therefore, A4.0 is important for mitigating several global concerns related to productivity, profitability, cost reduction and efficiency [11], including mitigating against climate change effects and allocating agricultural resources in a reasonable and ethical way [35]. Given this, there is an evident growing interest from the scholarly community towards the topic of digital agriculture [46].

A4.0 seems to be a more comprehensive and broader concept than PA, as it seeks to integrate all actors in agri-food production through a technological value chain. In this perspective, A4.0 goes beyond local and specific analyses to include all agricultural processes. Studies related to A4.0 address the development of decision support systems to integrate all members of the production chain [1], responsible innovation [47], real-time integration of IoT for agriculture [48], and the potential of A4.0 to address major societal issues associated with environmental sustainability (e.g., carbon emissions and exploitation of natural resources), social (e.g., impact on labor and food security) and economic sustainability (e.g., productivity, costs and agriculture as a subsistence economy) [11,32,49,50,51,52], among others. 

## 3. Materials and Methods 

We use the PICOC protocol and SciMAT software to perform a Bibliometric Performance and Network Analysis (BPNA) to achieve our goal. PICOC ensured the robustness and definition of the research criteria. At the same time, SciMAT helped identify the strategic themes, the thematic evolution structure and the main challenges and opportunities in the field of study.

### 3.1. PICOC Protocol

The PICOC protocol was used to define the research questions [53], the key terms and variants [54], and the inclusion and exclusion criteria for the selection of papers related to the field of study [55]. This method ensures the quality and reproducibility of the research with the lowest bias from the researchers. In this study, the process of the PICOC protocol was adapted, slightly modifying the search and analysis steps presented by Bruzza et al. (2017) and Silva et al. (2020) [55,56]. Table 1 shows the attributes according to the PICOC protocol.

With the support of the PICOC protocol, we defined three major research questions (RQ1–3):

**RQ1:** *What are the strategic themes related to Digital Agriculture?*
**RQ2:** *How has the thematic evolution of Digital Agriculture occurred over time?*
**RQ3:** *What are the main challenges and opportunities of Digital Agriculture?*

### 3.2. Network Analysis and Dataset

The search string, database, inclusion and exclusion criteria and bibliometric software used are present in Table 2. The documents identified through the execution of the PICOC protocol were inserted into the SciMAT software for BPNA. The search terms used were mentioned in previous research [11,57,58] to represent Digital Agriculture and cover more relevant, related studies.

**Figure 1:**
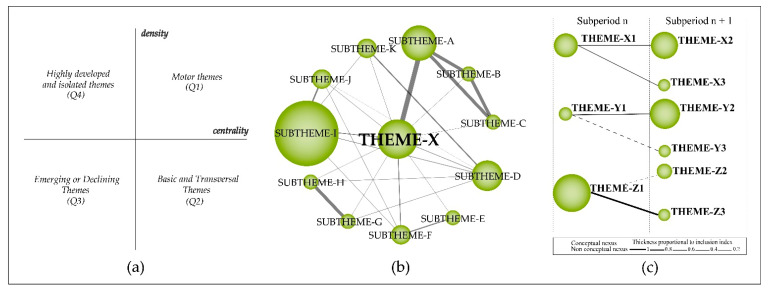
(**a**) Strategic diagram; (**b**) Thematic network structure; (**c**) Thematic evolution structure.

For bibliometric analysis and identification of strategic themes and the evolutionary map of the field, we used the SciMAT software developed by Cobo et al. (2012) [30]. We chose the SciMAT because it assists all stages of scientific mapping, from data preprocessing to the generation of maps of the field of study [30,52,59,60]. In addition, the SciMAT presents a robust preprocessing module, which ensures data quality [30]. With the support of the SciMAT, we plotted a strategic diagram and the intellectual network structure of the most important themes [61,62,63]. In addition, it was possible to generate an evolutionary diagram that presented the most important themes of the field of study distributed over time. Figure 1 illustrates an example of the strategic diagram (a), thematic network structure (b), and thematic evolution structure (c), where the size of the clusters represents the number of associated documents and the importance of the theme for the research field, and the thickness of the lines represents the strength of the relationship between the clusters [30,59,60]. The evolutionary map was created based on the equivalence index, where the solid lines (Figure 1c) indicate that the clusters share the main theme (main theme ∈ {thematic nexuses}). In contrast, dashed lines represent a connection between the non-main elements (main theme ∉ {thematic nexuses}), and the absence of lines means that the theme was discontinued from one period to the following period(s) [64].

**Figure 2:**
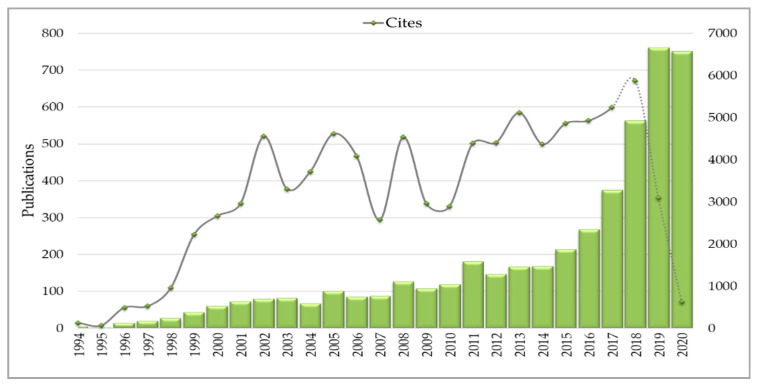
Publications over time (1994–21 September 2020).

We used the ISI/Web of Science (WoS) for analysis as it is an indexed database with a controlled quality of research [65]. The data were extracted from the database on 21 September 2020. Altogether, 4701 documents were exported from the Web of Science and seven duplicate documents were removed. Afterwards, 14,700 keywords related to the documents were identified and inserted into the SciMAT preprocessing step. Keywords with the same meaning were then grouped, such as ‘geostatistic’ and ‘geostatistics’, ‘internet of things’ and ‘IoT’, and meaningless or generic words were removed, totaling 13,935 clusters of words. After preprocessing, the data were inserted into the software in order to generate the strategic diagram of the field of study and later subdivided into three subperiods (1994–2011; 2012–2017; 2018–2020) to generate the evolutionary map. As the software does not generate this division, we separated the subperiods considering the prior literature. In this way, it was possible to identify from the first document published in the Web of Science (published in 1994) that discusses precision techniques and the field’s evolution until the emergence/introduction of Agriculture 4.0 in 2011 (1994–2011). The second subperiod (2012–2017) reflects the evolution of agriculture after the emergence/introduction of the Agriculture 4.0 concept and the use of new technologies in agriculture. Finally, the third subperiod (2018–2020) presents the most relevant themes emerging as of today and provides valuable insights into the future of the research field, since a huge amount of papers were published in this last subperiod (as pictorially shown in Figure 2).

After defining the subperiods, we generated the diagrams based on the keyword co-occurrence matrix with the SciMAT to identify the most important themes and their nodes with other clusters [66,67,68]. To determine the similarity between themes, data were then normalized using the Equivalence Index [69] and clustered based on the Simple Centers Algorithm (SCA) to create a network of relationships [70]. To make the diagrams, we reduced the data on a frequency reduction of eight times. We defined a maximum and minimum network of 12 and 3, respectively, to plot only the most important clusters of the field of study (for more information, see Cobo et al. (2012) [30]). The clusters were plotted on a two-dimensional diagram with four quadrants (Q1–4) (e.g., Figure 1a) whose horizontal axis represents the centrality of the themes and their number of connections with other themes, and the vertical axis represents the density of the connections between clusters [11,13,59,60]. In other words, clusters with high centrality tend to co-occur with many other themes, while clusters with high density have very dense co-occurrence links with specific themes. This analysis highlights the correlational breadth between two or more themes over time. The four quadrants are defined as follows:

- Motor themes (Q1): the upper right clusters are highly developed themes. The strong centrality and density represent the high number of associated documents and the many links between these themes and others. In this sense, motor clusters are the most developed and important themes in the field of study and are related to many other subjects/topics.
- Basic and transversal themes (Q2): clusters in the lower right quadrant are themes that have many relationships with other themes but whose relationships are weak. Despite these clusters’ great development and links with different themes, the low density indicates little capacity to sustain over time.
- Emerging or declining themes (Q3): they represent clusters with few and weak links with other themes, which may appear or disappear in the field of study. These themes need in-depth qualitative analysis by researchers to identify whether they are emerging or declining. Over time, these clusters may cease to be discussed or move to other quadrants.
- Highly developed and isolated themes (Q4): the upper left quadrant comprises of low centrality and high-density clusters. These clusters have few but strong links with other themes. The strong centrality of these themes shows their ability to prevail over time, to develop links with other clusters and, perhaps, to become motor themes.

The motor themes of the strategic diagram were explored to identify the relationships with other themes. In this way, it was possible to identify technologies, techniques and other widely discussed subjects/topics and the main challenges and opportunities of Digital Agriculture. 

As a last remark to the methodology adopted in the present study, we would like to stress two major points. First, as also previously mentioned, to the best of our knowledge, the present investigation is the first to perform a bibliometric analysis related to the field of Digital Agriculture. As such, this study should be intended as being of an exploratory nature, providing readers with a preliminary, though broad, comprehensive, and detailed overview of the existing scholarly literature covering the research area of Digital Agriculture. Therefore, we decided to focus on PA, not including other PA-related terms as keywords, and we did not attempt to explore its multi-faceted, complex nature and its sub-sectors (namely, PH, CEA, VF, PAF or Precision Aquaculture/Fisheries). These aspects warrant further ad hoc reviews (either in the form of systematic overviews or mapping/scoping reviews), based also on the findings of the present investigation.

## 4. Results

**Figure 3:**
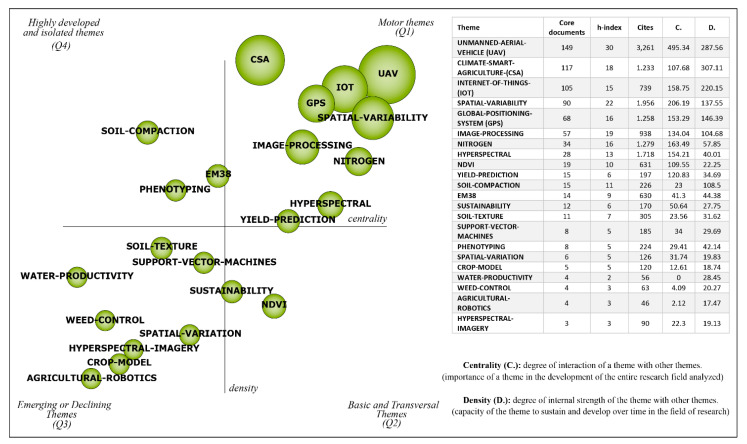
Strategic diagram and performance analysis.

**Figure 4:**
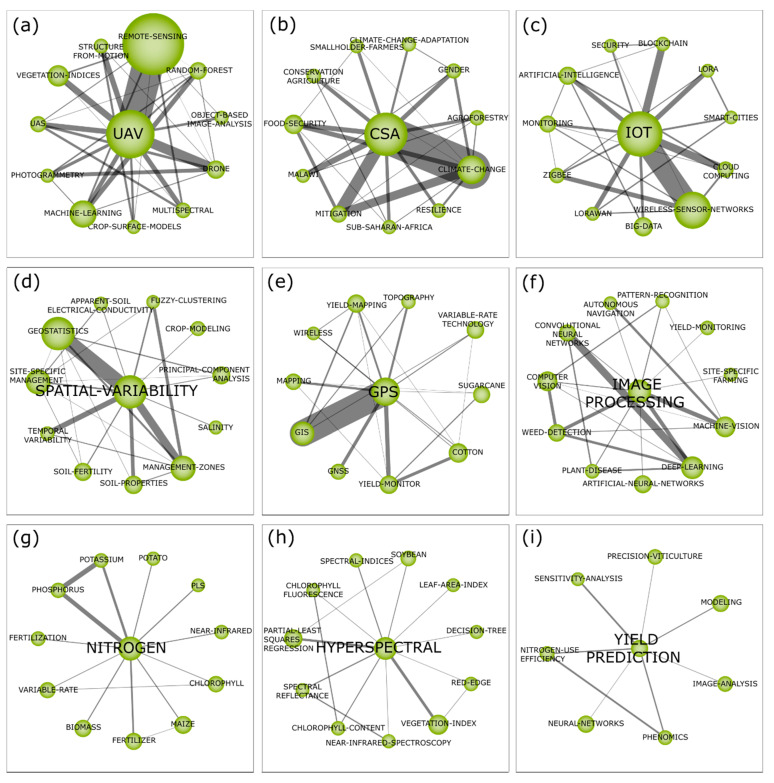
Thematic network structures. (**a**) UAV; (**b**) CSA; (**c**) IoT; (**d**) Spatial Variability; (**e**) GPS; (**f**) Image Processing; (**g**) Nitrogen; (**h**) Hyperspectral; (**i**) Yield Prediction.

**Figure 5:**
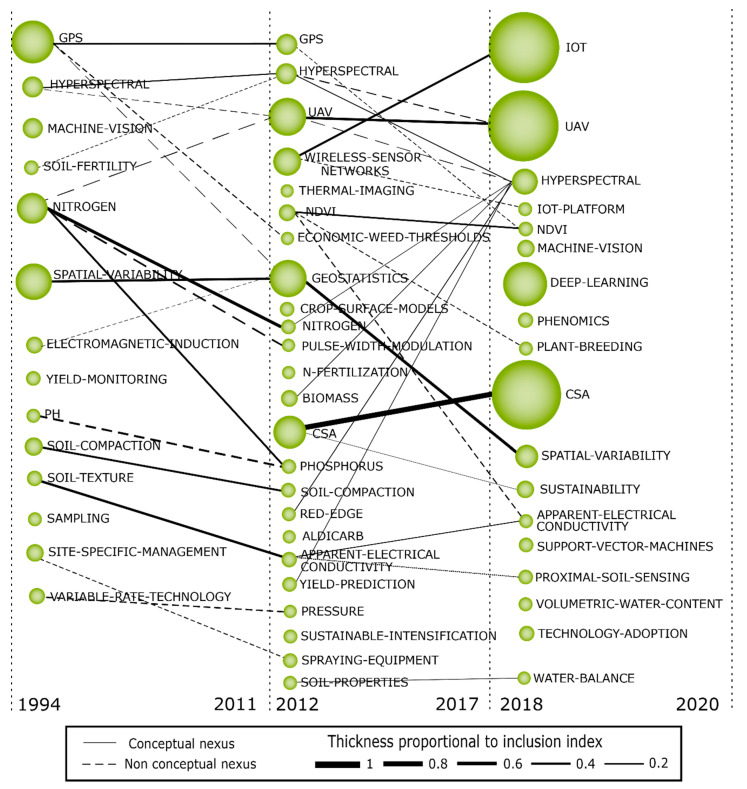
Thematic evolution structure.

To explore in-depth the DA field, we conducted a BPNA, which assesses DA-related scholarly performance in terms of productivity over time and main journals which published DA-related studies, the strategic themes (Figure 3), the thematic network structures of the motor themes (Figure 4), and the evolutionary map of the field (Figure 5). This thorough analysis enables us to understand the transformations in agricultural production techniques and their impacts on sustainability, organizations, and society as they have been explored in the existing scholarly literature, in terms of publishing trends.

### 4.1. Bibliometric Performance Analysis of Digital Agriculture

In 1994, the term Precision Agriculture started to be widely discussed in studies that evaluated the rate of application of inputs in crops [71], the spatial and temporal variability of yields [72,73,74] and the potential of precision techniques for conserving natural resources [75]. After the initial introduction and its subsequent use, the field of study has continued to grow, and new concepts have emerged over time. In 2011, with the emergence/introduction of Industry 4.0, the term Agriculture 4.0 came to have a broader emphasis and scope for adopting digital technology in agriculture and representing the creation of a value chain [11,32]. 

Figure 2 shows the number of studies published over time and highlights the importance and expansion of research. In 1994, only five articles related to Digital Agriculture were published, in 2017, the number reached 374 articles, and 762 studies were published in 2019. In 2020, the data collection date justified the decrease in publications (21 September 2020). The increase in the number of publications in recent years is warranted by the development and maturity the field is reaching and achieving and by the number of researchers who have made efforts to understand the impact of technology adoption in agriculture. Different aspects of a field of study can be understood and quantified through bibliometric analysis [76]. Uncovering critical elements, such as leading journals and the most productive authors, can help young researchers to identify important journals and seminal references in the field of study [77]. In this sense, Table 3 presents the journals and authors that published the most studies related to Digital Agriculture indexed in Web of Science from 1994 to 21 September 2020, based on the search string used. The most productive journal is the *Computers and Electronics in Agriculture* with 407 documents, followed by *Precision Agriculture* (231) and *Sensors* (187). The authors with the greatest number of studies related to the theme are Sudduth, K.A. with 46 documents, Lopez-Granados, F. (36) and Shearer, S.A. (31). It should be stressed that this list is just for guidance purposes and does not take into account the type of papers published (original article versus review/overview).

### 4.2. Strategic Diagram

The Strategic Diagram (Figure 3) presents 22 clusters distributed in four quadrants of the two-dimensional diagram, of which nine are motor themes (Q1), two are basic and transversal themes (Q2), eight are emerging or declining themes (Q3), and three are highly developed and isolated themes (Q4). The size of each cluster is proportional to the number of associated documents. The horizontal axis represents the links from one cluster to others (centrality), and the vertical axis demonstrates the strength of these links (density). The table in Figure 3 shows the core documents, h-index, number of cites, centrality (C), and density (D) of each theme. 

The ‘UAV’ (Unmanned Aerial Vehicle) cluster contains the largest number of associated documents. It is the most expressive theme in terms of h-index, number of cites and centrality, highlighting this cluster as one of the most discussed themes in the literature. The cluster ‘CSA’ (Climate-smart Agriculture) also has expressive core documents. It is the first ranked in terms of density, but its low centrality shows that the relationship between CSA and other themes is still underexplored. Other clusters that stand out are ‘IoT’ (Internet of Things), ‘SPATIAL-VARIABILITY’, ‘GPS’ (Global Positioning System), ‘IMAGE-PROCESSING’, ‘NITROGEN’, ‘HYPERSPECTRAL’ and ‘YIELD-PREDICTION’ (Figure 3). These motor themes (Q1) are explored in depth through the thematic network structures in the following subsection. 

In addition, ‘NDVI’ (Normalized Difference Vegetation Index) and ‘SUSTAINABILITY’ appear to be basic and transversal themes (Figure 3) and represent the concern for the sustainable development of agriculture. Other techniques appear related to crop management, such as ‘WEED-CONTROL’, ‘CROP-MODEL’, ‘SOIL-TEXTURE’, ‘PHENOTYPING’, ‘SOIL-COMPACTION’ and ‘WATER-PRODUCTIVITY’, and others are related to technologies, such as ‘HYPERSPECTRAL-IMAGERY’, ‘SUPPORT-VECTOR-MACHINES’, ‘EM38′, and ‘AGRICULTURAL-ROBOTICS’. Although some clusters represent specific analyses and techniques related to productivity and quality of agricultural production, the motor themes highlight the transformation expected for food production in the coming years. 

### 4.3. Motor Themes Related to Digital Agriculture

We explored the thematic network structure (Figure 4) of the motor themes presented in the strategic diagram (Figure 3). This analysis highlights the relationships of a cluster with others and provides an in-depth view of the field of research. The size of each cluster is proportional to the number of associated documents, and the thickness of the lines denotes the binding force between clusters.

#### 4.3.1. Unmanned Aerial Vehicle (UAV)

The cluster ‘UAV’ presents significant importance in the strategic diagram (Figure 3) due to its high degree of interactions with the other motor themes. The UAV corresponds to a pilotless aircraft, i.e., a flying machine operated with no humans (passenger/pilot) onboard [78]. Some previous literature reviews and meta-analysis explored the use of UAVs in agriculture, as in research by Lelong et al. (2012) [79], Singh and Frazier (2018) [80], Shahbazi et al. (2014) [81] and Eskandari et al. (2020) [82]. UAVs are low-cost and easy-to-operate technologies in agriculture [83] that can be adopted for spraying in fields [84], such as water precision irrigation. Given the technological advancement emerging in different countries, UAVs have gained more and more space and importance for smart farming [83,85]. Various sensors can be coupled to UAVs [85] associated with techniques, such as photogrammetry, Structure from Motion (SfM), multi-spectral and hyper-spectral images, and technologies, such as Machine Learning, LiDAR, and sensors, cameras and other hardware and software that contribute to monitoring the crop. This seems to justify the strong relationship between UAV and the subtheme ‘REMOTE-SENSING’, followed by the subthemes ‘MACHINE-LEARNING’ and ‘VEGETATION-INDICES’ among others (Figure 4a).

Figure 4a shows the relationship between UAV and Unmanned Aircraft System (UAS), while UAV focuses on the development and use of robots; UAS includes ground-based controllers, communication systems, payloads, electro-optical systems, radar, and software for aircraft vehicles. The sensors in the drones can generate vegetation indices, helping to monitor crop growth diagnosis and grain yield prediction [86]; disaster detection in environmental areas [87]; the existence of pests and pathogens in the crop; the analysis of the health and stress status of the vegetation; and the assessment of soil fertility for appropriate remote management and precise and reduced application of agrochemicals, driving sustainable agriculture [85] and increased productivity. New, sophisticated, cutting-edge techniques and approaches, such as machine learning, improve decision-making in the field of precision and digital agriculture, assisting and facilitating tasks like the prediction of nitrogen index of the crop through remote sensing from UAV [83,88]. Thus, farm-related data and information can be easily collected through drones, such as images [89], and can generate knowledge that machine learning techniques can process to make agriculture more precise and digital. For instance, Zhang and Zhao (2019) [90] used images of coastal soils captured through UAV to identify salinization levels and then proposed a method to improve the monitoring and management of the soil for avoiding the negative impacts of salinization on crop growth and production. Hence, this cluster highlights the central role of UAV, which, together with other emerging technologies, generates precision in agriculture and enables the interconnection among technologies for better decision-making, allowing farms to be smart.

#### 4.3.2. Climate-Smart Agriculture (CSA)

CSA (Figure 4b) is a concept related to the adoption of best agricultural production practices and methods for reorienting agriculture for environmental protection, food security and climate change issues [15,91]. The population increase, land and environmental degradation, and the negative impacts of the food system on the climate require new agricultural practices. Within this framework, CSA is an extremely necessary approach to find win-win solutions for the sustainable development of agriculture [92]. Climate change and the degradation of the planet’s biodiversity cause risks to the food supply, and the vulnerability of people who have agriculture as their primary source of subsistence [15]. To deal with this, the resilient agriculture model proposed by CSA seeks to create adaptive coherence between climate and agricultural practices [92]. To assist in the development of CSA, farmers embrace technological innovations that could make agriculture more sustainable. However, great governmental and academic incentives need to be dedicated to assisting the agricultural sector in sustainable development [91,92].

This high-density motor theme is related to sustainable agriculture, which shows its strong relationship with climate-related themes (e.g., ‘CLIMATE-CHANGE-ADAPTATION’ ‘CLIMATE-CHANGE’), environmental preservation (e.g., ‘CONSERVATION-AGRICULTURE’ ‘AGROFORESTRY’), organizations (e.g., ‘SMALLHOLDER FARMERS’) and discussions related to agricultural resilience, food security and the main challenges for organizations, especially for smallholders. Previous systematic reviews explored the role of CSA [91,93,94,95]. Related research discusses technological adoption [96], farmers’ adaptation to resilient agriculture [97], innovative economic models [98], emerging ways to increase the resilience of smallholder farmers [99], and the impact of CSA on food security [100], among other issues associated with resilient agriculture.

#### 4.3.3. Internet of Things (IoT)

The cluster ‘IoT’ (Figure 4c) has a high degree of interactions with the subtheme ‘WIRELESS-SENSOR-NETWORKS’, followed by ‘BIG-DATA’, ‘ARTIFICIAL-INTELLIGENCE’, ‘CLOUD-COMPUTING’ and others. Internet of Things can be defined as a grouping of technologies and infrastructures that, wirelessly connected, enable data mining from interconnected objects, as well as the monitoring and correct management of data [11,101]. It allows remote access and control of objects [102]. Thus, IoT has been achieving a prominent role in agriculture. Both the quality and quantity of production in the agricultural industry have been maximized through IoT [103], such as crop yield and efficiency [104]. IoT technologies, such as smart sensor networks, cameras, weather stations and smartphone applications, can improve crop data collection and decision-making for smart farm management [105].

There are many ways of adopting IoT in agriculture. Some authors have explored previous literature to understand IoT’s role, opportunities and challenges in agriculture [106,107,108]. Jin et al. (2020) [109] proposed a predictor based on IoT to accurately predict/forecast temperature and humidity in fields, allowing anticipated planning and control for sustainable precision agriculture. Jayaraman et al. (2016) [105] proposed an IoT platform capable of expanding data collection on soil, irrigation, fertilization and environmental conditions, generating relationships among data for forecasting crop performance and boosting smart farming. Relating IoT to blockchain, Hussain (2019) [110] developed a model capable of helping in the migration from traditional to smart agriculture. Tekinerdogan (2018) [3] used IoT to create a portable agricultural sensor network system, which collects data (temperature, light intensity, and soil moisture content) utilizing sensors and a microcontroller to generate databases that can be used through Big Data based analytics for better crop management. These IoT actions can be further enhanced and maximized when combined with artificial intelligence-based technologies, which facilitate the management of data collected from different objects and sources in the field [102]. In addition, connectivity on Digital Agriculture through IoT becomes efficient and effective when based on cloud computing [111]. Thus, regardless of the applications, IoT actions in agriculture require investments in cloud computing to facilitate the collection, storage, access, and management of integrated data. Therefore, this cluster emphasizes the importance of IoT as a link among different technologies, tools, equipment, and machines that jointly improve precision agriculture and, consequently, lead to digital agriculture.

#### 4.3.4. Spatial Variability

The cluster ‘SPATIAL VARIABILITY’ (Figure 4d) is a motor theme that presents strong interactions with geostatistics and management zones and contributes to monitoring soil properties at agricultural sites. The analysis of the spatial variability of soil attributes was one of the first precision techniques associated with crop management and helps farmers to know chemical and physical characteristics of the soil through sampling and analysis that identify soil variations such as nutrients, fertility, salinity, electrical conductivity, pH, organic matter, phosphorus, and other important variables/parameters. Geostatistics techniques such as kriging, variogram analysis and interpolation reveal the heterogeneity of the soil. These analyses allow us to understand intrinsic factors, such as soil formation and relief, substances, organisms and climate, and extrinsic factors, such as management practices, and fertilization rate, among others. 

Therefore, this cluster is linked with increasing efficiency and crop productivity. The analysis of spatial variability is, indeed, a key factor for increasing food production [112] and farmers’ economic revenues [113]. The mapping of soil heterogeneity has contributed to enhance soil data and to make geostatistics become a fundamental tool in agricultural management [114]. Some studies show that farmers visualize the information of DA made by georeferenced analysis [115]. Despite its advantages, spatial variability analyses often have high costs that make it difficult for small farmers to use them. Future research needs to explore the improvement of these techniques to increase the expansion of its use.

#### 4.3.5. Global Positioning System (GPS)

Through georeferencing, GPS was one of the first big steps towards Digital Agriculture. The cluster ‘GPS’ (Figure 4e) is a motor theme that presents strong interactions with the subtheme Geographic Information Systems (GIS). Other subthemes, such as ‘YIELD-MONITOR’, ‘YIELD-MAPPING’ and ‘VARIABLE-RATE TECHNOLOGY’, also have prominent links with this cluster. Thus, the integration of GPS with other smart technologies, such as GIS and remote sensing, can boost farming productivity and cost efficiency [116]. GPS corresponds to a navigation system that, using satellites, identifies an object’s exact position and velocity around the world and can guide directions [117]. Agricultural activities, such as seeding and plowing, can only be precisely conducted using vehicle navigation accuracy [118]. 

In previous research, Si et al. [118] proposed a method to enhance the accuracy of the steering angle of agricultural vehicles by combining GPS with the low-cost Micro-electromechanical System (MEMS), which improves DA. Palaniswami et al. [119] argue that the joint adoption of GPS and GIS technologies can improve sugarcane cultivation in coastal lowland ecosystems, making production more precise and sustainable. Khosro (2018) [120] developed a strawberry yield-monitoring picking cart, equipped with a real-time kinematic global positioning system (RTK GPS), different types of sensors, a microcontroller, and an inertial measurement unit to improve the synchronization of the carts with robots in harvesting and creating yield mapping of strawberry productivity. Commonly, yield mapping on farms is performed using GPS and variable-rate technology for boosting planters’ performance [121]. In addition, Thrikawakaa et al. [122] demonstrate that applying variable-rate technology through GPS in agricultural vehicles improves the water content in fields and reduces the application of fertilizers. In addition, previous literature reviews introduce the conceptualization and potential of GPS (for more information see, Rivero et al., 2021 [123]; Peng et al. (2017) [124]). Thus, this cluster highlights the role of GPS as a leading technology capable of improving DA’s effectiveness through its interconnecting capacity with other smart technologies. Emerging technologies combined with scientific knowledge make it possible to use the strategic geographical location of agricultural production, helping to make precise and targeted decisions.

#### 4.3.6. Image Processing

The cluster ‘IMAGE PROCESSING’ (Figure 4f) presents strong linkages with agricultural management tools (e.g., ‘MACHINE VISION’ and ‘COMPUTER VISION’) that provide data for the farmer’s decision-making. Previous literature reviews explored advanced control strategies and image processing techniques in Digital Agriculture (see Ngugi et al. (2021) [125], Syeda et al. (2021) [126], and Sohail et al. (2020) [127]). Zhao et al. [128] used machine vision to exploit color images to detect immature green citrus. The use of video processing (i.e., machine vision) also was analyzed by Sabzi et al. [129] to classify potato plants and three types of weed. In this regard, machine vision was also revised by Wang et al. [130], combined with image processing as relevant tools for ‘WEED DETECTION’. 

Machine learning algorithms also are linked with image processing and can assist ‘DEEP LEARNING’ in agriculture [131]. ‘ARTIFICIAL-NEURAL-NETWORKS’ is an example of the Artificial Intelligence method [132] to create new forms to solve complex agricultural problems based on big data. Through image processing, several outputs can be processed by machine learning and can be shown in apps for smartphones and computers [133].

#### 4.3.7. Nitrogen

The cluster ‘NITROGEN’ (Figure 4g), like the ‘HYPERSPECTRAL’ and ‘YIELD PREDICTION’ clusters, has no stronger relations with some themes than the other clusters. As an important nutrient for agricultural production, this cluster is related to crop management (e.g., ‘MAIZE’ and ‘POTATO’), and fertilizers (e.g., ‘PHOSPHORUS’ and ‘POTASSIUM’). The link between ‘NITROGEN’ and these themes comprises several fields of studies, such as nitrogen deficiency [134], nutrient management [135,136], nutrient loss [137], nutrient efficiency [138], and nutrient monitoring [139]. Therefore, the technical agronomic backdrop is preeminent in this cluster. In addition, this theme is strongly linked to efficiency in applying nitrogen and other applications of variable-rate technology [135,136,140].

#### 4.3.8. Hyperspectral

Several technologies are being developed and tested to analyze plants, soil and crop variables, such as optical sensors, three-dimensional (3D) imaging, fluorescence imaging, thermography, multispectral and hyperspectral imaging [141]. The ‘HYPERSPECTRAL’ cluster (Figure 4h) shows that these analyses are standing out in agriculture, mainly because hyperspectral imaging is a more advanced technique than traditional multispectral imaging and allows the identification of more details of the target features [142]. Hyperspectral images are used as an efficient alternative for collecting and processing data and creating an electromagnetic spectrum of agricultural fields, being a valuable tool for monitoring, analyzing, and providing more accurate detection of spatio-temporal variations in crop physiological and morphological characteristics [142,143], such as soil attributes and soil degradation. Some previous research explored the use of multispectral and hyperspectral techniques in the field of Digital Agriculture, focusing on high-resolution applications, and plant disease detection, among others [142,144,145].

Despite their great potential, hyperspectral images are still little used outside the academic context, but this is changing through low-cost and mini-sized airborne hyperspectral technologies that are being used by farmers [142]. This difficulty occurs due to the complexity of technologies and algorithms, and the need to store and transfer data collected by hyperspectral analysis devices [146]. The significance of the ‘HYPERSPECTRAL’ cluster shows the volume of analyzes performed using maps and images of crops. The clusters associated with this theme demonstrate analyses related to vegetation indexes and characteristics (e.g., ‘VEGETATION-INDEX’, ‘CHLOROPHYLL-CONTEXT’, ‘CHLOROPHYLL-FLUORESCENCE’ and ‘LEAF-AREA-INDEX’). Previous research used hyperspectral remote sensing to enable measurement of grapevine drought stress [147], identification of pests and diseases [148], and assessment of seed germination [149]. 

#### 4.3.9. Yield Prediction

Yield prediction is historically used in agriculture for farmers to plan inputs, labor, profitability and other issues related to agricultural productivity. The cluster ‘YIELD-PREDICTION’ (Figure 4i) in Digital Agriculture represents the evolution of predictive analysis on crop yields, which in the past was carried out by the knowledge of farmers, and is now carried out with emerging technologies [98]. In this way, preharvest crop yield prediction helps farmers to make better and faster decisions related to planting, fertilization, irrigation, pruning and the harvesting of plantations [150]. It also contributes to the entire supply and consumption chain, and allows the projection of future scenarios for food production [151]. 

This cluster appears related to be related to few themes, but we can highlight ‘MODELING’, ‘IMAGE-ANALYSIS’ and ‘NEURAL-NETWORKS’ that are used to map and model crop data and information for future predictions. Previous research related to crop yield prediction used different techniques and technologies to collect and analyze data, such as Neural Network Techniques [100], Unmanned Aerial System Imagery [152], Airborne Optical Sensors [153], Artificial Neural Networks [154], Machine Learning [155,156,157], Hyperspectral Imagery and Ensemble Learning [158], among others.

### 4.4. Thematic Evolution Structure

The evolutionary map presents the themes that have stood out over time. The map was divided into three subperiods to show the most important themes according to the evolution of the field of study. The first subperiod presents the main themes studied since the first mention of the term PA in 1994 in the ISI/Web of Science until the emergence of the Agriculture 4.0 concept in 2011. The second subperiod covers the years 2012 to 2017 and highlights the terms widely discussed since the appearance of A4.0. The last subperiod (2018–2020) highlights the most current and relevant themes in the literature in the previous years. Figure 5 shows the evolutionary map of the field of study, the size of the clusters is proportional to the number of associated documents, while the thickness of the lines represents the relationship between the themes. Continuous lines represent conceptual nexus and dashed lines have non-conceptual nexus.

#### 4.4.1. First Subperiod (1994–2011)

In this subperiod (1994–2011) (Figure 5), we identified the first studies that mentioned Digital Agriculture through discussions about the use of technologies to increase productivity, deal with environmental variability and environmental conservation of agribusiness [71,72,73,74,75]. This subperiod contains studies that used hyperspectral maps to analyze vegetation indices [159,160], imaging techniques [161] and geostatistical analysis [162]. Besides, Figure 5 shows that technologies started to be explored at this time, such as ‘GPS’, ‘HYPERSPECTRAL’ images, and specific analyses of soil and plants, such as ‘SOIL-FERTILITY’, ‘NITROGEN’, ‘ELECTROMAGNETIC-INDUCTION’, ‘PH’, ‘SOIL-COMPACTION’ and ‘SOIL-TEXTURE’. These themes show that few technologies were discussed and most of the themes were associated with crop variability. These themes prove that the discussions related to Digital Agriculture mainly represent the use of technologies such as georeferencing to analyze variability associated with soil and plants. 

In this subperiod, the first discussions on agroecosystems were developed [163] to create strategies to mitigate against environmental damage. It is important to note that this subperiod was one of the most important for discussions related to sustainability, as research was paving the way for other in-depth studies aimed at exploring the development of resilient agriculture and the sustainable development of agribusiness. In addition, many studies in this subperiod predicted that Digital Agriculture would be the future of agribusiness [164,165,166,167,168,169]. 

#### 4.4.2. Second Subperiod (2012–2017)

The second subperiod (2012–2017) (Figure 5) was characterized by a greater number of documents associated with the consolidated PA motor themes and with emerging A4.0 ones, as new technologies started to be discussed as the application of UAV [170], wireless sensor networks and NDVI. The presence of clusters still related to the first subperiod, such as ‘NITROGEN’, ‘HYPERSPECTRAL’ and ‘GPS’, demonstrates that some themes have remained strong over time. In addition, new themes emerged, such as ‘GEOSTATISTICS’ with a significant number of documents, and ‘SUSTAINABLE-INTENSIFICATION’ and ‘CSA’ associated with the development of resilient and sustainable agriculture, demonstrating the further expansion and growth of the field of study [171,172,173].

Between 2012 and 2017, the term A4.0 gained space and came to be widely discussed in the literature to portray the creation of a digital value chain in agriculture. Although many terms are discussed in this subperiod, few of them stand out in the volume of associated documents and interrelationships with themes from other subperiods. Only the ‘GEOSTATISTICS’ cluster has a strong relationship with the ‘SPATIAL-VARIABILITY’ clusters of the first and third subperiods, highlighting the high potential of geostatistics for analyzing crop variability. In addition, ‘CSA’ has evolved to become one of the main themes of the third subperiod. It is important to note that some themes have evolved over time; however, the small size of most clusters shows that the field of study was still under development. 

#### 4.4.3. Third Subperiod (2018–2020)

The third subperiod (2018–2020) (Figure 5) presents the most recent themes related to Digital Agriculture. Three clusters stand out with a large volume of associated documents. They are ‘IoT’, ‘UAV’ and ‘CSA’, which indicates that the use of emerging technologies and sustainable development are currently the most important concerns of researchers in the primary sector. The ‘IoT’ and ‘UAV’ clusters show the potential and adaptability of small sensors for any agricultural scenario, whether in large crops or in mountainous areas that are difficult to cultivate [11], while the ‘CSA’ cluster shows that concern for the sustainable development of agriculture continues to grow and is characterized as one of the most important discussions in the arena of smart agriculture [97,100]. In addition, discussions related to deep learning and machine vision represent major technological adoptions in the field of agriculture, and themes like ‘APPARENT-ELECTRICAL-CONDUCTIVITY’ [174], ‘WATER-BALANCE’ [175] and ‘PROXIMAL-SOIL-SENSING’ [176] show that the concern with analyses of plants and soil remains extremely important for the digital transformation of agriculture. 

A broad look allows us to see the evolution of the field of study and the main themes discussed over time. The third subperiod also produces insights on future trends, because some themes stand out from the others and are anticipated to remain of paramount importance. Besides, other themes have emerged only in the last subperiod and may represent emerging themes that can be further explored in depth in the coming years. The growth in the number of documents in the field of study (Figure 2) and discussions associated with technological adoption in agriculture (Figure 4) demonstrate the commitment of researchers to the development of the primary sector and its importance for the sustainable development of society and organizations.

## 5. Challenges and Opportunities

### 5.1. Main Challenges

Discussions related to UAV, spatial variability, yield prediction and crop model (Figure 3) show that there are many challenges associated with the adoption of emerging technologies in agriculture, such as variations in soil, climate, sustainability, cultivated species and producer size. Government investments, export volume, agricultural and consumer culture are also factors that directly impact the way food is grown and produced, and the innovative efforts and investments dedicated to production. Developed and emerging economies have different types of governmental investment for agricultural production, and some are developing projects aimed at the digital transformation of agriculture in countries, like China [177], Brazil [178], Mexico [179], Thailand [180,181] and others. Despite the investment of many countries in digital agriculture, the transformation is still in its infancy compared to the technological adoption in the manufacturing and service sectors. Technologies widely used in other sectors such as big data, Machine Learning, blockchain, simulation/mathematical modeling and augmented reality are still little explored in agriculture. Figure 3 shows that only Internet of Things and UAV are prominent technologies in the arena of agriculture. 

Among the main challenges of digital technology, technological adoption is among the characteristics and peculiarities of emerging technologies, which are often complex for the low level of technical knowledge of smallholder farmers [45]. The most significant volume of agricultural production is from developing countries, and most of these farmers are small producers with low levels of education. Under these circumstances, even language can become a barrier to technological innovation, since most technologies are developed in English, a language not spoken by small producers in developing countries [31]. This barrier for small producers favors large agribusiness companies that have workers trained to use technologies and apply them to increase large-scale production quality. In addition, there is a major digital infrastructure problem in many developing countries, which are gradually developing ways to bring digital devices and applications at low cost to small producers to increase profitability and production quality. This concern is evident in the relationship between smallholder farmers and CSA (Figure 4b), which also appears to be related to discussions of gender and female management in agriculture.

The motor theme of the field of study, the Internet of Things, appears related to security and blockchain (Figure 4c), highlighting that technological advances bring also complex data security and privacy issues. Concerns about information, confidentiality and data integrity are major challenges that concern farmers [182]. In highly technological scenarios, these concerns increase the efforts of researchers and practitioners in the search for better encryption and security techniques to reduce the risks of cyber-attacks and ensure data security and privacy [183]. According to Zhang et al. (2020) [184], farmers are highly concerned about data security and the physical requirements for equipment and practical tools for data protection and distributed systems for agricultural management. Hussein (2019) [183] reviews the requirement of the computational infrastructure necessary for cloud storage to be effective and secure. This and other studies highlight the concern with agile software development, data security, confidentiality, and reliability to guarantee fair competition, differential privacy, economic development, sustainable innovation, and social entrepreneurship in agriculture. 

In this sense, two major strands of challenges can be considered: the first strand is about technical challenges, such as the mastery and the adaptation of technological tools to direct them in the best possible way for optimal gains. These challenges are evident through technologies that are subthemes of thematic network structures, such as Machine Learning and Deep Learning, drones, cloud computing, big data, blockchain, Artificial Intelligence, artificial neural networks, computer vision, and others (Figure 4). The second strand is related to sustainability issues, such as the continuous world population growth and global warming (mainly due to the greenhouse effect, or gas emissions) and their harmful effects on agriculture [185]. Discussions related to these challenges are represented by subthemes, such as food security, conservation agriculture, climate change adaptation, agroforestry, vegetation indices, resilience, and mitigation.

Moreover, it is crucial to keep in mind that, although many authors discuss the technological potential for promoting sustainable development, other authors discuss the paradox of sustainability [186] by raising questions about the contradictions between economic growth and sustainable development that mainly negatively affect the low-income populations [187]. In the same perspective, the technological paradox regards the lack of evidence about technological benefits in terms of productivity and sustainability of organizations and society [188]. These controversies between authors show that discussions about these paradoxes in agriculture are not yet sufficiently developed and addressed, and that future research should explore this topic in-depth [52].

### 5.2. Opportunities for Research and Practice

The primary sector is crucial for human survival and is full of challenges. In this scenario, the technological potential can be exploited to meet the food needs of the human population without damaging the environment [52]. The number of studies associated with sustainable agriculture shows that technological adoption can develop resilient agriculture that integrates the various (economic, environmental, and societal) pillars. Previous research related to the motor theme of CSA explored the adaptation of agricultural methods to develop adaptive agriculture for plantations, landscapes and forests while addressing climate issues and food security [91,92]. Regenerative and circular agriculture explores ways to avoid using chemicals and to rather utilize methods to develop natural processes to protect the soil, the environment, and people through the production of healthier foods [189]. Figure 4 highlights this through subthemes, such as food security, soil fertility and related properties/topics. These subthemes also help us identify the main crops where the technology is being implemented and discussed in the literature, such as sugarcane, cotton, potatoes, maize, and grape (viticulture). Thus, the formulation and adoption of new technologies based on the circular economy principles (reduce, reuse, recycle) can boost the sustainability, digitalization, and integration of agriculture with other links of the agribusiness value chains, a promising opportunity for further advancement of the field. 

Advances in agricultural production are crucial for increasing production and improving logistics performance and traceability of agricultural data in the coming years [52]. Although big machines favor agricultural monopolies, small technologies, such as sensors, actuators, and drones can be developed at a lower cost and with features to assist small farmers in collecting and analyzing crop variability data [11]. Other technologies, such as smart grids can assist in the development of integrated energy networks that facilitate the management of crops at lower costs [190]. Digital Agriculture depends on technological progress, and a viable path for future research concerns strategic foresight [191] and analysis of innovation capabilities [192,193] to assist in the development of technological innovations in smart agriculture. Our results are presented in the evolutionary map (Figure 5) with the emergence of themes such as thermal imaging, red edge, spraying equipment, NDVI, and technology adoption.

Digital Agriculture can promote and foster changes, revolutions, transformations, and gains, both for society and sustainability, as well as for economic agents through the improvements in scale and efficiency of the digitization of agriculture. Within this framework, if drones and IoT are more connected to companies and businesses, CSA has an important impact on inclusive and sustainable agriculture. The existing scholarly literature presents several techniques and technological solutions aimed at ensuring environmental protection and economic and social sustainability (see Figure 5, e.g., hyperspectral, thermal imaging, spatial variability, proximal soil sensing, and CSA, among others), which represent both technological and sustainable revolutions in the primary sector. This may motivate a cycle of sustainable production and consumption to transform the planet and society. According to Ofori and El-Gayar (2020) [31], the main drivers for technological adoption in agribusiness are related to governance, policies, trade opportunities, effects on human capital, employment opportunities and environmental protection. In this perspective, traditional agriculture is no longer sufficient to meet the demand for food, quality, sustainability and safety of the environment, and a new paradigm is needed, namely the technological paradigm [194]. 

To create a technological and sustainable culture, agriculture depends on the engagement of various members of society, such as universities, farmers, companies, the community and the government. In this sense, the topic of creation of an innovation ecosystem has been little explored in the literature and presents an opportunity for future research. The participation of universities is crucial for developing technologies and research aimed at modeling and developing methods and techniques that assist farmers in managing agricultural processes. Governmental programs and financing can help in the digital transformation of agriculture in developing countries, promoting the production and consumption of sustainable products, less waste and awareness of recycling. In this perspective, innovation ecosystems can be explored in-depth for the digital and sustainable transformation of the primary sector. The adoption of new technologies enables the transformation of value chains into digital ecosystems, potentially integrating organizations and stakeholders, benefiting each participant in the ecosystem [195]. As in industries, in agriculture, value ecosystems can directly impact economic performance and business agility. To do this, national innovation systems can assist in transferring knowledge between industries and universities [196,197], facilitating the development of digital innovation.

In addition, the number of startups focused on agribusiness is growing, and they are developing technologies at a lower cost that meet the needs of small and medium farmers. All of this can help, in particular, small producers, who are responsible for a large portion of the world’s population’s food production, and who are affected the most by the challenges of agricultural revolutions. Technologies, such as big data, facilitate the collection and analysis of large volumes of data, and IoT, through its small sensors and actuators, can be used in any type of setting/environment. In addition, cybersecurity can be exploited to address farmers’ fear of data protection and security. Altogether these approaches can facilitate the digital transformation of agriculture in different scenarios and for different sizes of agricultural producers.

## 6. Conclusions

In this research, we presented the strategic themes and the scientific evolution structure of Digital Agriculture. With the PICOC protocol and the SciMAT software it was possible to map and identify the strategic themes of the field of study, which are mainly associated with technologies and sustainable development, such as IoT, UAV, GPS, spatial variability, and CSA. In addition, the evolution map divides the literature into three subperiods of time, listing the most important themes at each stage of development of digital transformation in agriculture. The main challenges associated with digital technological adoption were identified, mainly related to the complexity of technologies, agricultural culture and adaptation, data security/safety/integrity and environmental issues [198,199]. In addition, we identified the main opportunities of technological adoption and research in agribusiness that are strongly related to creating a technological and smart value chain to increase the productivity, quality, and profitability of agricultural production, while promoting and ensuring economic, societal, and environmental sustainability. Thus, we believe to have covered a major gap in the existing scholarly literature related to the evolution of the digitalization of agriculture and its strategic themes over time.

### Limitations and Further Research

Despite its contribution, methodological rigor and high-quality, as well as transparency, and reproducibility, this research has limitations that should be properly acknowledged. This study explored only documents from the Web of Science database, and we analyzed only the thematic structures of the motor themes, and as such other themes should be addressed in future works. It is important to keep in mind that agriculture directly impacts socioeconomic conditions and environmental sustainability. Thus, a more comprehensive qualitative analysis could be realized in terms of the implications of Digital Agriculture for sustainability and societal development. The role of emerging technologies to promote food security and the impact of food production on society and organizations need to be explored. Future research can explore in-depth agricultural management practices focused on CSA, sustainable and lean agriculture to identify and analyze sustainable production paths. In addition, scenarios and frameworks for smart agriculture implementation should be explored. Future research could map the field of study, focusing on specific techniques, applications, and technologies such as Big Data, Artificial Intelligence, blockchain, Internet of Things, autonomous robots, NDVI, yield prediction, image processing, etc. Future studies could explore each theme and subtheme not covered in this research to discover hidden and emerging themes in the field of study, such as those related to subsectors of PA, like PH, CEA, VF, PAF and Precision Aquaculture/Fisheries. Moreover, further additional analyses could be performed, stratifying findings based on the specific study design of the investigation (original research, overview/review, etc.) and gradating/assessing its strength of evidence. Innovation ecosystems need also to be examined by involving organizations, government, and universities to integrate and digitize processes. Future works can also explore technological and sustainability paradoxes to identify the real potential of technologies in agriculture and the challenges related to their contradictions to better understand whether human decisions about food production are moving in the right direction or are causing an ecological collapse unrecoverable on the planet.

## Figures and Tables

**Table 1:** Steps defined according to the PICOC protocol.

Attributes	Description
Population (P)	Define keywords, search terms and variants related to Digital Agriculture.
Intervention (I)	Define the document’s inclusion and exclusion criteria.
Comparison (C)	The approach was a BPNA.
Outcome (O)	The outcomes are the strategic diagram and the evolution map of Digital Agriculture.
Context (C)	The future of Digital Agriculture is discussed through the main challenges and opportunities.

**Table 2:** Quality assessment criteria.

Quality Assessment	Description
Search string	(“agriculture 4.0” OR “digital agriculture” OR “digital farming” OR “smart agriculture” OR “smart farming” OR “precision agriculture” OR “precision farming” OR “agri-food 4.0”)
Database	ISI/Web of Science (WoS)
Inclusion and exclusion criteria	Only documents with the search terms present in the title, abstract or keywords Related or applicable to digital agriculture Only documents in English No time restriction/filter
Bibliometric software	Science Mapping Analysis Software Tool (SciMAT)

**Table 3:** Most productive journals and authors. It should be stressed that this list is just for guidance purposes and does not take into account the type of papers published (original article versus review/overview).

Most Productive Journals	Doc.	Most Productive Authors	Doc.
Computers and Electronics in Agriculture	407	Sudduth, K.A.	46
Precision Agriculture	231	Lopez-Granados, F.	36
Sensors	187	Shearer, S.A.	31
Remote Sensing	171	Schmidhalter, U.	21
Applied Engineering in Agriculture	101	Ribeiro, A.	21
Transactions of Asabe	87	Bareth, G.	17
Biosystems Engineering	71	Miao, Y.X.	16
Geoderma	70	He, Y.	15
